# Hospital Meetings, &C.

**Published:** 1898-05-28

**Authors:** 


### The Institutional Workshop.

### HOSPITAL MEETINGS, &C.

### THE LONDON HOSPITAL.

Dinner at the Mansion House.

A dinner was given by the Lord Mayor at the Mansion House on Wednesday evening, 18th inst., to further the interests of The London Hospital. The Duke of Cambridge, who is president of the hospital, was the principal guest, and the company, which numbered over two hundred, included the Duke of Grafton, Viscount Falkland, Viscount Knutsford, Lord Masham, Lord Monk Bretton, Sir George Faudel-Phillips, Bart., the Hon. Alban Gibbs, M.P., the Hon. Evelyn Hubbard, M.P., the Hon. Sydney Holland (chairman of the hospital), the Hon. W. F. Massey-Mainwaring, M.P., Mr. Spencer Charrington, M.P., Sir M. M. Bhownaggree, M.P., K.C.I.E., Mr. Hudson Kearley, M.P., Mr. J. H. Robertson, M.P., the Hon. Lionel Holland, M.P., Sir J. Whittaker Ellis, Bart, Admiral Colomb, Sir Joseph Sebag Montefiore, Sir Trevor Lawrence, Bart, Sir Christopher Furness, Sir Henry Burdett, K.C.B., Sir Robert Giffen, K.C.B., Sir J. T. Ritchie, General Sir W. Stirling Hamilton, Sir Frederick Young, K.C.M.G., Mr. Alfred Beit, Mr. F. D. Mocatta, Mr. William Lidderdale, Mr. Julius Wernher, Mr. Alderman and Sheriff Green, Mr. Sheriff Dewar, the Masters of the Salters', Distillers', and Turners' Companies, Mr. John H. Hale, Mr. F. C. Carr-Gomm, and Mr. W. J. Soulsby, C.B.

Letter from Mr. Balfour.

After the loyal toasts, the Lord Mayor read the following letter which he had received from Mr. A. J. Balfour:—

"10, Downing Street. My dear Lord Mayor,—It is with very great regret that I find myself prevented from taking any part in the dinner over which you are to preside in aid of the funds of The London Hospital. The special work which that hospital has to do in connection with the East-end renders it a most deserving object of public benevolence, and it will certainly not be creditable to the richest city in the world if any serious difficulty is found in providing funds in support of an institution on which so many of the London poor are necessarily dependent for medical and surgical assistance. Wishing your dinner every success, I beg to remain, yours very truly, Arthur James Balfour." (Applause.)

"Success to the Hospital."

The Lord Mayor then proposed "Success to The London Hospital." He was proud of the fact that, although he was presiding over what he would term a charity dinner, he was not called upon to invite his guests to give subscriptions. He was bound, however, to tell them what was required. He had recently visited the hospital, and had been much struck by the very great difference there was between some of the wards and others. Some were perfectly charming, others were not. On asking the reason for this, he had been told it was owing to want of funds. He then asked himself the question, "Is it right that this noble institution should suffer for want of funds?" He replied, "No, it is not; it must not, and it shall not." (Cheers.) The accommodation for the staff was altogether inadequate and insufficient, both in quantity and in quality; there was not a single isolation-room in the hospital, and its kitchens were quite unworthy of it. The hospital was situated, perhaps, not in the poorest part of London, but certainly not in the richest. It was placed amidst the working classes who did so much good for this Empire, and for that reason alone it was, he thought, deserving of support. (Applause.)

The Duke of Cambridge, in responding, said he wished to express to the Lord Mayor his sense of the advantage he (the Lord Mayor) had done to the hospital in allowing that dinner to be held in the Mansion House of the City of London. (Applause.) The London Hospital was established 150 years ago. The buildings and the requirements of those days were very different from those of the present time, and they were endeavouring to bring into the old building all the requirements of present-day science. To do that funds were urgently needed; and, although the City of London had done a great deal for the London Hospital, it could not do all that was necessary. It was the general public, also, who must respond to the appeal. The London Hospital had done a great deal in progressing with the age, but more remained to be done. Amongst other things which were necessary was a second operating theatre. At the present time the daily requirements were such that many of the operations had to be performed in the wards. The nursing staff, too, should be increased, and their accommodation made better. Financial assistance was, however, essential before these improvements could be carried out, and he trusted the appeal which had been made would be quite successful. (Applause.)

The Case for The London Hospital.

The Hon. Sydney Holland proposed "The Health of the Lord Mayor." He first of all thanked the Lord Mayor for what he had done for The London Hospital, not only on behalf of all those present that evening, but on behalf of the committee of the hospital, and on behalf of the splendid staff, medical and surgical, with whom the House Committee worked without the slightest friction and with the greatest cordiality. (Applause.) The way in which he had got the Lord Mayor to exert himself on behalf of the hospital was this. One day, when wandering through the City seeking whom he might devour for the purposes of the hospital, he found himself opposite the Mansion House and was seized with an inspiration. He said to himself that if his cause was a good one (and he was sure it was) why should he not place it before the leading citizen of London. He did so, with the result that the Lord Mayor consented to break through the ordinary procedure of the Mansion House and to give a dinner in aid of The London Hospital. That evening they were all his guests. That action on the part of the Lord Mayor was known throughout the East-end of London, and it would always be remembered there with great thankfulness. (Applause.) As had been announced, no subscriptions would be asked for at this dinner; but though he (the speaker) was forbidden to ask for subscriptions *at* the dinner, he was not prevented from asking for donations *after* the dinner—(laughter)—and if there was any gentleman present who felt inclined to help them he would be most happy to see him in the vestry after the service. (Laughter.) Previous speakers had told them of the great alterations which it was necessary to make in the hospital. The making of those alterations would necessitate the closing of wards containing 100 beds for a space of three years. It would, however, be impossible to do without those beds, and therefore, amongst other things, some £2,000 was required to build a temporary hospital to take those poor patients whilst the alterations were being carried out. If any gentleman would give this sum he would be doing a kindness to no less than 5,000 poor people during the course of those three years. (Hear, hear.)

The previous speakers had not exaggerated one whit the needs of the hospital. The London Hospital was face to face with the most serious crisis that it had ever had to surmount in the 150 years of its existence, and it would be impossible for him, without being wearisome, to set out before them that evening all its needs. Here was the case for the hospital in a nutshell. It was the only general hospital for a district containing over a million and a half of people; it was within seven minutes' walk of the richest City of the richest country in the world; it was the largest voluntary hospital in England; and yet still it was cramped in every direction for want of means. It was not cramped for the want of a splendid staff—they had attending the hospital certainly as good a staff as any hospital in England. (Applause.) It was not cramped for want of willing workers—they had on their House Committee thirty gentlemen, who were most active, and who worked for the hospital with heart and soul. (Renewed applause.) It was not cramped for want of patients, but it was cramped in every direction for want of means to carry on the work there was to do. They had had to sell out of their investments no less than £30,000 in the last two years; £40,000 in the last three. They required, to carry on this hospital, no less a sum than £70,000 a year.

He had heard people say, "Oh, we can't do anything; the thing is too vast for us. It is impossible." Why impossible? It never had been impossible for their ancestors to keep up that hospital. They had done their duty to the East-end poor of their day. Was the present generation to say that they could not do the same? Was this the country, was this the place in this country, to complain of a work being too big? Surely, at a moment when England was richer than she ever had been they were not to hear Englishmen say, "We cannot keep up this work, not because it is not doing enough, but because it is doing too much good." He could not and would not believe that. Why, its very vastness was one of its means of usefulness. The very vastness of the hospital gave to their doctors and surgeons the opportunities of seeing and treating diseases of all kinds in a way and in numbers that a small institution could not do. The very vastness of the hospital was of use, not only to the staff, but to the 350 students who studied at The London Hospital medical school, and who would in time become the doctors and surgeons of England. (Applause.) The very vastness of the hospital, therefore, was a reason for its being helped largely. (Renewed applause.) But although the hospital was vast, it really was not large enough for their purpose. Owing to want of space, they had to treat the patients who came to them too hurriedly. Owing to want of beds, they were unable to treat the patients they ought to treat, and whom they were determined to help. (Applause.) Owing to want of space, they were obliged to turn out of the hospital almost every day poor men, poor women, and poor children who were only half cured, and they were obliged to do this in order to make room for people who were urgently in need of immediate attention. The chairman had referred to the isolation block at the hospital. He (the speaker) would go farther than the chairman had, and would say that he would not put a dog of whom he was fond into the isolation block of the London Hospital at the present moment. Then, too, the operating theatre was far too small for their needs. Was it not intolerable that they should prepare people for an operation, and that afternoon after afternoon they should have to tell them that the theatre was full, and that they could not be operated on that day? Just let his hearers try and realise how much mental and physical suffering was involved in cruelty such as that. And all this—the result of the limited room and the limited funds at their disposal—spelt cruelty to the poor of the East-end. The raising of the funds necessary to alter all this was by no means an easy task, and the life of the chairman was not altogether a happy one. There were, however, some incidents which had made his lot happier. Some little time ago a gentleman went over the out-patient department. Afterwards he came to him (the speaker) and said, "I will give you £25,000 to alter and enlarge that department." (Applause.) Another gentleman made most careful inquiries into the number of operations which were performed under anæsthetics at The London Hospital. He found that no less than 8,000 operations under anæsthetics took place last year, and that, owing to there being only one operating theatre, a great many of these operations had to be done in the wards. The result was that that gentleman had given him (the speaker) £10,000 to build a new operating theatre. (Loud applause.) Both these gentlemen wished to remain anonymous. Those were deeds which gladdened the heart of any chairman.

Apart, however, from all sentimental considerations—he was speaking that night to business men and thinking men—it was not actually economy for Londoners not to do their utmost to promote the interests of a hospital such as The London. It was bad economy to keep the bread-winner of the family away from his work when everyone knew that every day he was away from his work and his pay the powers of recuperation of his family were getting less; but it was worse economy to have to send him back to his work only half cured. It was bad economy, too, to let children grow up maimed by injury, dwarfed by illness, to face the tussle of life when they could supply the means of sending them out strong. It was miserable economy to weaken the fibres of the citizens of this great metropolis by not doing the best they could for its poorer inhabitants. (Applause.) Nothing touched him more than the fact that these poor people accepted the situation in which they were placed with resignation and patience. They knew that, except through the kindness and the charity of richer people, many illnesses meant to them perpetual disablement; and yet he had never seen any unrest amongst them when it concerned themselves, but only when someone they dearly loved was affected. These poor people for whom he was appealing had very few joys. Their object in life was not to see how much enjoyment they could get out of it, nor how to make their homes more comfortable and their surroundings more enjoyable. No, all the energies of the poor for whom he was speaking seemed to him to be concentrated upon considering how it was possible to drag through life at all. They had two joys, however, which they shared with the richest in the land. One was the love for father, for mother, for husband, for wife, and for children. That joy could not be altered, could not be added to. The other joy was health, and although his hearers could not possibly ensure to them good health, humanly speaking they could if they liked—if they would pay for it—make it easier for them to keep it. (Loud applause.) Was it very much, then, for him to ask those present with all the earnestness at his command to give to these poor people, who with pleading eyes looked up to them, all the chances of recovery from illness that they possibly could? Might he conclude with the words which always appealed to him so deeply, "Man, if you cannot look down with tenderness, how can you look up with hope?" (Loud applause.)

The Lord Mayor announced that so far the result of the joint appeal of the House Committee and the Lord Mayor had been the collection of £39,045 in donations, and £4,250 in new annual subscriptions, besides the £35,000 received by the Hon. Sydney Holland from two anonymous donors for the out-patient department and the operating theatres.

The proceedings shortly afterwards terminated.

### ROYAL FREE HOSPITAL.

The seventieth anniversary festival dinner of the Royal Free Hospital was held at the Hotel Cecil on Friday, 20th inst. The Marquis of Dufferin and Ava (president of the institution) was in the chair, and amongst those present were the Marchioness of Dufferin and Ava, Lord Justice Collins and Lady Collins, Mr. Justice Bruce (chairman of the committee) and Lady Bruce, Sir William MacCormac, Bart., and Lady MacCormac, Sir Henry Burdett, K.C.B., Sir Gerald Fitzgerald, K.C.M.G., Sir John and Lady Hutton, Sir Richard H. W. Wyatt, Sir George B. Bruce, and Mr. and Mrs. Burt.

In proposing the loyal toasts, the Chairman said that the Prince of Wales had recently turned his attention to the difficult question of putting the maintenance of the hospitals of this country on a proper footing. It must be a matter of satisfaction to all to see that His Royal Highness had shown by his example how incumbent on all was the duty of ministering to the wants and necessities of the sick. (Applause.)

In proposing "Prosperity to the Royal Free Hospital," the Marquis of Dufferin and Ava said that he was not going to make any appeal to the feelings of his hearers. This meeting, however pleasant, was a business meeting, and not an occasion for a display of sentimental charity. There was no doubt that the present day was more humane than its predecessors. The wants, the sufferings, and the trials of their fellow creatures occupied a greater space in their thoughts than they ever had before. Society had become aware of its shortcomings in the past, and was very sensible of its duties in the present and the future. As a consequence, the efforts which were being made in every direction to relieve human suffering were many. Probably there was no phase of misfortune which was not to a certain degree provided for, but notwithstanding the multiple character of charitable endeavour the best they did fell miserably short of what was required. They were like mariners aboard a leaky ship. They were perpetually labouring at the pumps, but the water was constantly gaining upon them. The rapid rate at which charitable institutions were increasing was adding a new difficulty as well, for institutions now frequently came into competition with one another. The butter might extend over a larger surface of bread, but it was spread much thinner. As a result institutions were nowadays compelled to blow their own trumpets, and to show how their claims on the support of the public were more imperative than other establishments of which nothing but good should have been said. As president of the hospital he had no hesitation in saying that there was no hospital, nay, there was no institution either in London or from one end of Great Britain to the other which was more worthy of the support of the public than the Royal Free Hospital. Although the hospital had been continuously making additions and improvements for some time past yet still there were many things which were still required. In the first place, many improvements were wanted in the wards, additional heating apparatus and lifts were necessary, and the sisters and nurses must be made more comfortable. (Applause.)

Lord Justice Collins, in proposing "The Committee of Management and the Weekly Board," said that people living in London, seeing day after day the great structures of our hospitals, came to regard them as permanent features in the ordinary life of this great City, and as a consequence but few people troubled to find out how they came into existence, or how they were managed and organised. It never occurred to them how complicated an organisation a hospital must be.

Mr. Justice Bruce and Mr. Charles Burt responded. The latter said that the object of the dinner was not merely to collect money, but it was intended to try and induce people to take a personal interest in the affairs and conduct of the Royal Free Hospital. Referring given to the dinner in aid of the London Hospital by the Lord Mayor, he said that although the Royal Free was not a great hospital with 800 beds, but only a modest one with 170, its affairs were carried on with as much economy and with as great efficiency as the larger institution. The London Hospital spent £70,000 a year and treated 11,000 in-patients last year. That was to say it expended £6 7s. per patient. The Royal Free spent £12,000 and treated 2,148 in-patients last year, or an expenditure of £5 12s. 6d. per patient. The amount required at the present time was £10,000, of which £3,000 was needed for providing separate bedrooms for their nurses, £800 to £900 for two lifts, and £5,000 for necessary improvements in the wards.

Other toasts followed.

In responding to the toast of "The Visitors," which was proposed by Mr. H. J. Allcroft, Sir Henry Burdett said that the Royal Free Hospital had always been a great hospital for accidents, it had always been a free hospital, and it had always been famed for the amount of personal service which had been contributed to its work, both by its staff and by its management. It further had enjoyed a good reputation for its kindness to and success with its patients, and it was one of the few hospitals of London which did not wait for hospital reformers before it instituted an almoner to look after its out-patient department, in order that it might attach to free relief the guarantee that those who unworthily attempted to abuse that free relief should find it impossible to enter its doors. There was another attribute of the hospital, and that was its modesty: but he was inclined to think that the hospital had suffered from too much modesty. The Royal Free was the hospital of all hospitals which the women of London ought to take under their especial charge and care, as it was the one hospital in London which made it possible for women to gain an English medical degree. (Applause.)

Sir William MacCormac, Bart., proposed "The Health of the Chairman," who suitably responded, and the proceedings terminated.

A sum of £3,000 was announced as having been collected through the dinner.

### THE ALEXANDRA HOSPITAL.

The annual meeting of the governors of the Alexandra Hospital for Children with Hip Disease was held on Thursday, the 19th inst., under the presidency of Mr. W. H. Whitfield. The Chairman moved the adoption of the report, which conveyed the welcome intelligence that the income of the year enabled the committee, after making provision for all payments, to show a small balance of £10 on the right side. The receipts, amounting in all to £2,661, included annual subscriptions of £800, donations £450, and special subscriptions for the maintenance of cots £611. This last source of income, however, shows a decrease, and the hope is earnestly expressed that new subscribers will come forward to take the place of those who have been reluctantly compelled to withdraw their assistance. The festival dinner in connection with the building fund produced nearly £3,000, the total amount of this fund, according to the report, being £5,654. There is also a further amount of £1,742 realised by the sale of the Bournemouth property, which will be available for the building fund when required. Pending the rebuilding, premises for the continuation of the work have been secured at 34, Guilford Street. The hospital has 68 beds—60 in London and 8 in their convalescent home at Painswick. During the year there were 168 children under treatment. Of these 57 were discharged cured, 27 were improved, 2 died, 2 were discharged as incurable, 5 were transferred to other hospitals, 22 to convalescent homes other than Painswick, and 59 remained under treatment on September 30th. The number of out-patients was 290, and the total attendances amounted to 1,703. In passing a cordial vote of thanks to the visiting ladies, it was mentioned that many of them undertake the active education of children placed in the hospital.

### NATIONAL HOSPITAL FOR THE PARALYSED AND EPILEPTIC.

Mr. E. Silver, who presided at the annual meeting of this institution, held on Thursday, the 19th inst., had a satisfactory report to introduce to the governors and subscribers. The year 1897 was again one of progress for the hospital. During the year the total receipts from all sources reached the sum of £26,686, of which £17,649 was expended for general purposes, while of the balance £920 was applied to endowment, £1,871 allotted to the convalescent home building fund, £4,466 to the commemoration fund, and £1,402 to the pension fund. In last year's report the Board referred to the need of carrying out certain important works at a cost of £10,000, and proposed to raise this sum by way of commemoration of Her Majesty's Diamond Jubilee. More than half the amount asked for had been received, and in thanking all who have responded to the appeal the Board remark that the rearrangement of the out-patient department, the improvement of the accommodation for nurses, the provision of an additional open space for the patients, and the further extension of the hospital site with a view to the future are urgent needs, only to be met by means of a special fund, of which the sum asked for would be a very valuable nucleus. The hospital received £750 as an annual contribution from the Prince of Wales's Fund, and £752 10s. from the fund as a special donation. Coming to the work of the institution, the in-patients during the year numbered 1,001, and the average number of beds occupied 163; these totals being lessened by reason of the beds at Finchley not being available during part of the year. The cost of each occupied bed amounted to £76 18s. 5d., and of each individual in-patient to £11 18s. 10d. The out-patients numbered 5,939, and the total attendances 36,974, the cost of each out-patient being approximately 5s. 0½d. It is pointed out that the great importance of the figures relative to the attendances of out-patients is found not so much in the numbers as in the fact that a large and increasing proportion represents persons sent at the recommendation of medical practitioners, and from other hospitals for an authoritative diagnosis of obscure and difficult cases, thus affirming in a practical way the views expressed by distinguished authorities as to the value of the hospital as an institution where the patient will obtain an opinion "beyond which there is no appeal," and where if his case is curable he will have the best means science can afford towards the restoration of his health. The charity has sustained a great loss in the death of Dr. J. S. Ramskill, the senior member of the medical staff whose services extended over a period of nearly thirty years. An extra appointment having been made two years back, no vacancy is caused by Dr. Ramskill's death. The report was unanimously adopted, and the usual votes of thanks passed.

### VICTORIA HOSPITAL FOR CHILDREN.

Mr. Martin Ridley Smith presided at the annual meeting of this institution, held at the hospital on Thursday, the 19th inst. The accounts show that the total receipts for the year, including £1,000 realised by the sale of India Stock, amounted to £9,541; and the total expenditure, inclusive of £1,289 to meet the deficit at the convalescent home at Broadstairs, to £8,069. The number of patients admitted during the year was 894, of whom 753 were cured or relieved. Of the remaining cases, 41 were discharged on account of some infectious disease, which rendered their removal necessary. Eighty-seven patients died. Of these deaths 66 occurred in infants under two years of age, and in many cases took place within 48 hours of their admission. In the out-patient department there were 56,022 attendances, 17,719 being new cases. The number of patients admitted to the convalescent home at Broadstairs was 445. The average stay in the hospital was 15 days, against 14 in the previous year; and the daily average number of beds occupied 58, as against 59. The death-rate per cent. works out at 9·2, as compared with 10 8. In referring to the fact that a decrease of eight is shown in the number of in-patients, the Chairman made a most important statement. They had, he said, been harassed by several outbreaks of infectious diseases in the wards, and though their drainage, after repeated tests, had been found to be as perfect as modern science could make it, their ventilation was not of so perfect a character. The house was an old one, and had not been designed for a hospital. Recently their medical committee recommended the reduction of the number of beds by 10, and this had reluctantly been adopted in the hope of keeping the hospital free of infection. The hope had not been fulfilled, and personally he had come to the conclusion that, taking the unsuitability and age of the structure into consideration, they should face the difficulty by the erection of a new building on the ample ground at their disposal. This matter had not yet been officially considered by the committee, but in his opinion the step must be taken, and he hoped to have the sympathy and aid of the governors towards carrying out the scheme. There was another matter he desired to mention. After grave deliberation the committee had decided to request the temperance and trades councils of Chelsea and Battersea to discontinue their annual demonstration on behalf of the institution, since the custom had given offence in several quarters. It was always a delicate and difficult matter to decline loyal and enthusiastic service, but they had represented to the councils that the interests of the hospital would best be served by a discontinuance of the custom. The councils, all honour to them, had loyally acquiesced in this suggestion, and they had now been invited to nominate a number of honorary governors. When the communication was made to their friends at Battersea the arrangements of the latter were so far completed that they had to go on with this year's demonstration. In this connection it is interesting to note that the demonstration referred to have during the last 15 years contributed nearly £3,000 to the revenue of the hospital. The report was unanimously adopted.

NORTH LONDON HOSPITAL FOR CONSUMPTION.

The Duke of Cambridge presided at the festival dinner in aid of the North London Hospital for Consumption, which was held at the Hotel Cecil on Thursday, the 19th inst. Amongst those present were: The Earl of Haddington, Lord William Seymour, Sir J. Savory, Bart., M.P., Lord Robartes, Lieutenant-General Bateson, Sir Henry Burdett, K.C.B., Colonel Hans Hamilton, Mr. E. Brodie Hoare, M.P., Colonel George Grant Gordon, and Mr. B. A. Lyon (chairman of committee). After the loyal toasts the Duke of Cambridge proposed "Prosperity to the North London Hospital for Consumption." The hospital, he said, was capable of containing 80 beds, but owing to want of funds only 60 had so far been opened, and he would urge on all the great necessity for supplying the money to enable the institution to work at its full strength. The working men of the district in which the hospital was situated gave whatever they could to support it, and he thought that in itself was a proof that the public at large had confidence in those that managed it. (Applause.)—Mr. B. A. Lyon, in responding, referred to the Prince of Wales's Fund. He said he was afraid that in the division of that great fund there had been a disposition to overlook the claims of the small hospitals. Although he recognised the paramount claims of the great hospitals yet in their degree he thought that the small special ones did a work which the great institutions could not do, for it would be impossible for the latter to treat special diseases such as consumption or cancer. He therefore hoped that those who administered that great Fund would not overlook hospitals like his own, even though they had not in their wards the 100 beds which had been set up as a standard.—This elicited a response from Sir Henry Burdett, who said that Mr. Lyon and his hearers must remember that in the first year of the existence of the Prince of Wales's Fund it was impossible to visit all the hospitals in London. That was why only hospitals with 100 beds constantly occupied were awarded grants other than those awarded on the Hospital Sunday Fund basis. It would, no doubt, be a consolation to Mr. Lyon to know that a special visiting committee had now been appointed, and that during the next few months all the hospitals of London would be inspected. He had no doubt that the distribution of this year's fund would be done with due regard to the work accomplished and its efficiency. — The subscription list amounted to £2,130.

THE HOSPITAL FOR WOMEN, SOHO SQUARE.

The annual meeting of the Hospital for Women was held at the hospital on Thursday, May 19th. In the absence of the Duke of Westminster, Mr. Victor A. Williamson took the chair. After paying a tribute to the memory of the late Duchess of Teck and to the late Sir Rutherford Alcock, K.C.B., who was chairman of the Board of Management, he went on to say that the financial position of the hospital occasioned them serious alarm. They were £3,000 in debt, and from that day the annual interest would amount to £120. All hospitals had cycles of fat and lean years. In their fat times, when in one year they had received £17,000, they had discharged liabilities to the extent of £18,000 and had rebuilt a certain portion of the cottages occupied by some of the patients and the nurses. There were those who had objected to spending the money on bricks and mortar. Their justification, however, was that the cottages not only were unsafe but possessed every attribute that was undesirable in a hospital. Although, the chairman went on to say, the fortunes of the hospital had never been at so low a financial ebb, there had never been a year in which so much work had been done. For 20 years they had kept all its wards open, and he trusted that a generous public would not allow them to be compelled to close them now. Lastly, in a short time the authorities hoped to publish the report of a sub-committee now sitting upon a new development of their work. All the large hospitals possessed convalescent homes to which to send their patients, and as they had about £400 in hand, balances from the two Samaritan funds—enough to furnish a small house if one could be found in a suitable locality, they were desirous of following the good example, and in due time to come before the public for a small contribution to carry their idea into practice. The report was then seconded by Mr. Edgar C. Harvie, and adopted unanimously. The retiring third of the committee of management were re-elected. The names of new governors were added to the list, and the usual vote of thanks to the chairman was cordially given. There were present: Captain G. A. Webbe, Mr. Charles Dunbar Buller, Mr. Sigmund Neumann, Lady Frances Turner, Lady Juliana Walker, Lady Elizabeth Knox, the Hon. Mrs. R. Gathorne Hardy, and others. During 1897, 720 patients were admitted as in-patients. The death-rate was 2·9 per cent. The ordinary expenditure was £6,583, extraordinary £85. The total ordinary income was £3,943, and the extraordinary includes a loan of £2,000 from the bankers and the sale of bank stock to the amount of £1,000. An annual sum of £2,000 is needed to make the income and expenditure balance.

